# The minimum number of examined lymph nodes was 24 for optimal survival of pathological T2-4 gastric cancer: a multi-center, hospital-based study covering 20 years of data

**DOI:** 10.1186/s12885-023-11138-0

**Published:** 2023-09-21

**Authors:** Lulu Zhao, Fan Zhang, Fuzhi Jiao, Xiadong Zhou, Penghui Niu, Xue Han, Wanqing Wang, Xiaoyi Luan, Mingyan He, Quanlin Guan, Yumin Li, Dongbing Zhao, Jidong Gao, Yingtai Chen

**Affiliations:** 1 National Cancer Center/National Clinical Research Center for Cancer/Cancer Hospital, Chinese Academy of Medical Sciences and Peking Union Medical College, Beijing, China; 2 Lanzhou University Second Hospital, Lanzhou, China; 3 The First Hospital of Lanzhou University, Lanzhou, China; 4 Gansu Provincial Cancer Hospital, Lanzhou, China; 5 National Cancer Center/National Clinical Research Center for Cancer/Cancer Hospital & Shenzhen Hospital, Chinese Academy of Medical Sciences and Peking Union College, Shenzhen, China

**Keywords:** Gastric Cancer, Examined lymph nodes (ELNs), Gastrectomy, Prognosis

## Abstract

**Introduction:**

The current National Comprehensive Cancer Network (NCCN) guidelines recommend that at least 16 lymph nodes should be examined for gastric cancer patients to reduce staging migration. However, there is still debate regarding the optimal management of examined lymph nodes (ELNs) for gastric cancer patients. In this study, we aimed to develop and test the minimum number of ELNs that should be retrieved during gastrectomy for optimal survival in patients with gastric cancer.

**Methods:**

We used the restricted cubic spline (RCS) to identify the optimal threshold of ELNs that should be retrieved during gastrectomy based on the China National Cancer Center Gastric Cancer (NCCGC) database. Northwest cohort, which sourced from the highest gastric cancer incidence areas in China, was used to verify the optimal cutoff value. Survival analysis was performed via Kaplan-Meier estimates and Cox proportional hazards models.

**Results:**

In this study, 12,670 gastrectomy patients were included in the NCCGC cohort and 4941 patients in the Northwest cohort. During 1999–2019, the average number of ELNs increased from 17.88 to 34.45 nodes in the NCCGC cohort, while the number of positive lymph nodes remained stable (5–6 nodes). The RCS model showed a U-curved association between ELNs and the risk of all-cause mortality, and the optimal threshold of ELNs was 24 [Hazard ratio (HR) = 1.00]. The ELN ≥ 24 group had a better overall survival (OS) than the ELN < 24 group clearly (P = 0.003), however, with respect to the threshold of 16 ELNs, there was no significantly difference between the two groups (P = 0.101). In the multivariate analysis, ELN ≥ 24 group was associated with improved survival outcomes in total gastrectomy patients [HR = 0.787, 95% confidence interval (CI): 0.711–0.870, P < 0.001], as well as the subgroup analysis of T2 patients (HR = 0.621, 95%CI: 0.399–0.966, P = 0.035), T3 patients (HR = 0.787, 95%CI: 0.659–0.940, P = 0.008) and T4 patients (HR = 0.775, 95%CI: 0.675–0.888, P < 0.001).

**Conclusion:**

In conclusion, the minimum number of ELNs for optimal survival of gastric cancer with pathological T2-4 was 24.

**Supplementary Information:**

The online version contains supplementary material available at 10.1186/s12885-023-11138-0.

## Introduction

Gastric cancer is a significant cause of cancer-related mortality, ranking fifth globally, and is the sixth most common type of cancer worldwide [1]. In China, roughly 70% of patients with newly diagnosed disease present with localized disease and may have the opportunity to undergo radical resection. In such patients, examined lymph nodes (ELNs) are a crucial prognostic determinant. Current National Comprehensive Cancer Network (NCCN) guidelines recommend that at least 16 lymph nodes should be examined for gastric cancer patients to reduce staging migration [2, 3]. However, accumulating evidence suggests that extended lymphadenectomy can be performed safely and provides a survival advantage [4–7]. As a result, there is ongoing debate regarding the optimal management of ELNs for gastric cancer patients.

Previous studies have attempted to identify a node threshold for theoretical oncologic benefit and survival advantage. Mirkin et al. [8] have investigated 1036 pathological N0 patients with gastric cancer who received neoadjuvant therapy, and found a survival benefit with 30 nodes examined. Even so, it needs to be considered that neoadjuvant therapy has the potential to downstage positive lymph node burden [9]. In addition, Smith et al. provided support that examining as many lymph nodes as safely feasible during gastrectomy for gastric cancer [6]. Brenkman et al. evaluated a Dutch cohort and showed that high number of ELNs was associated with prolonged survival in elderly patients [10]. With respect to the number of ELNs, Hu et al. confirmed that pathological N3 patients with > 31 ELNs exhibited superior prognostic utility using the Surveillance, Epidemiology, and End Results (SEER) database [11]. Furthermore, a published study ascertained that the optimal ELN threshold was 30 nodes for pathological N2 disease and 40 nodes for pathological N3 disease within the SEER cohort [5]. However, given known differences in eastern and western gastric cancer presentation and management, the conclusions above may not be suitable for Chinese population. Another concern is that an extensive lymphadenectomy is believed to increase surgical morbidity without providing an expected survival improvement due to the additional surgical trauma. Therefore, there needs to be an optimal threshold for the extent of ELNs, which can provide a survival benefit while reducing postoperative morbidity.

Given these considerations, we utilized restricted cubic spline (RCS) analysis to determine the minimum number of ELNs that should be retrieved during gastrectomy in order to optimize survival outcomes for patients with gastric cancer. This analysis was conducted using data from the China National Cancer Center Gastric Cancer (NCCGC) database. Additionally, we assessed the prognostic performance of the identified optimal ELN threshold in comparison to the previously recommended threshold of 16 ELNs, using the Northwest cohort sourced from the regions in China with the highest incidence of gastric cancer.

## Methods

### Data source and study population

**Fig. 1:**
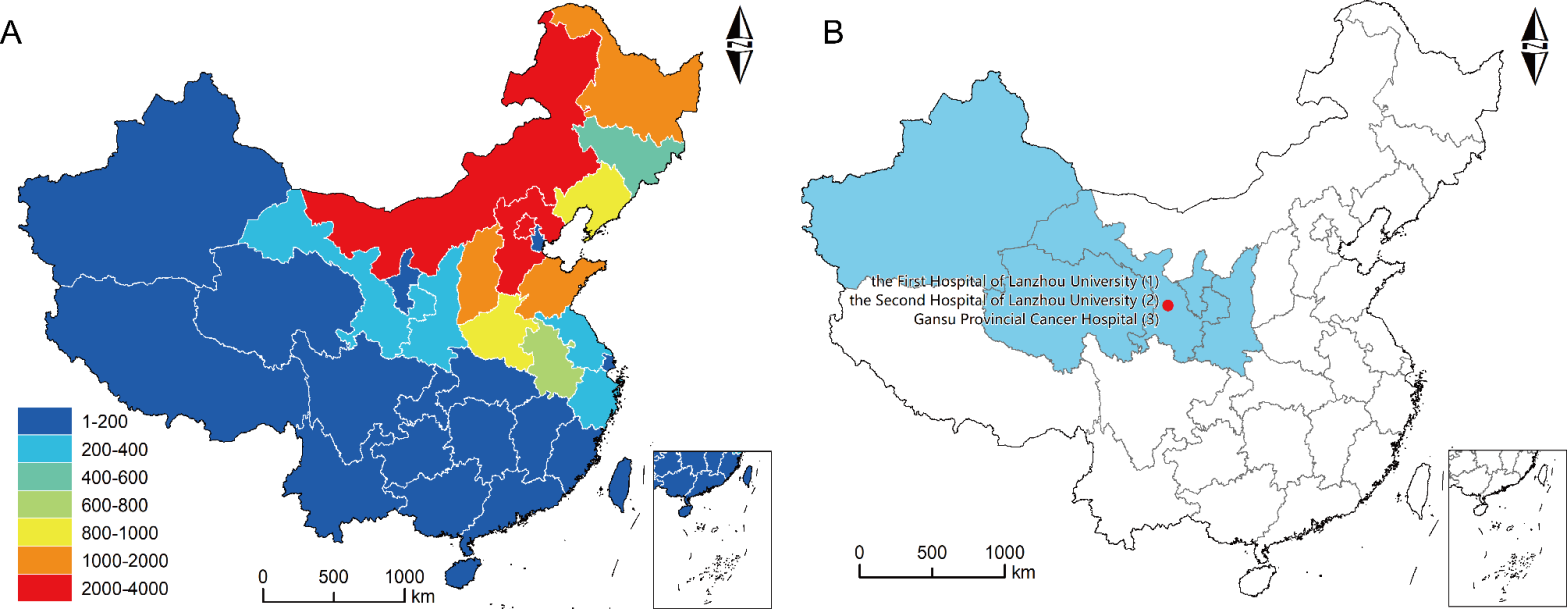
The geographical locations of gastric cancer patients of (**A**) NCCGC cohort and, (**B**) Northwest cohort

**Fig. 2:**
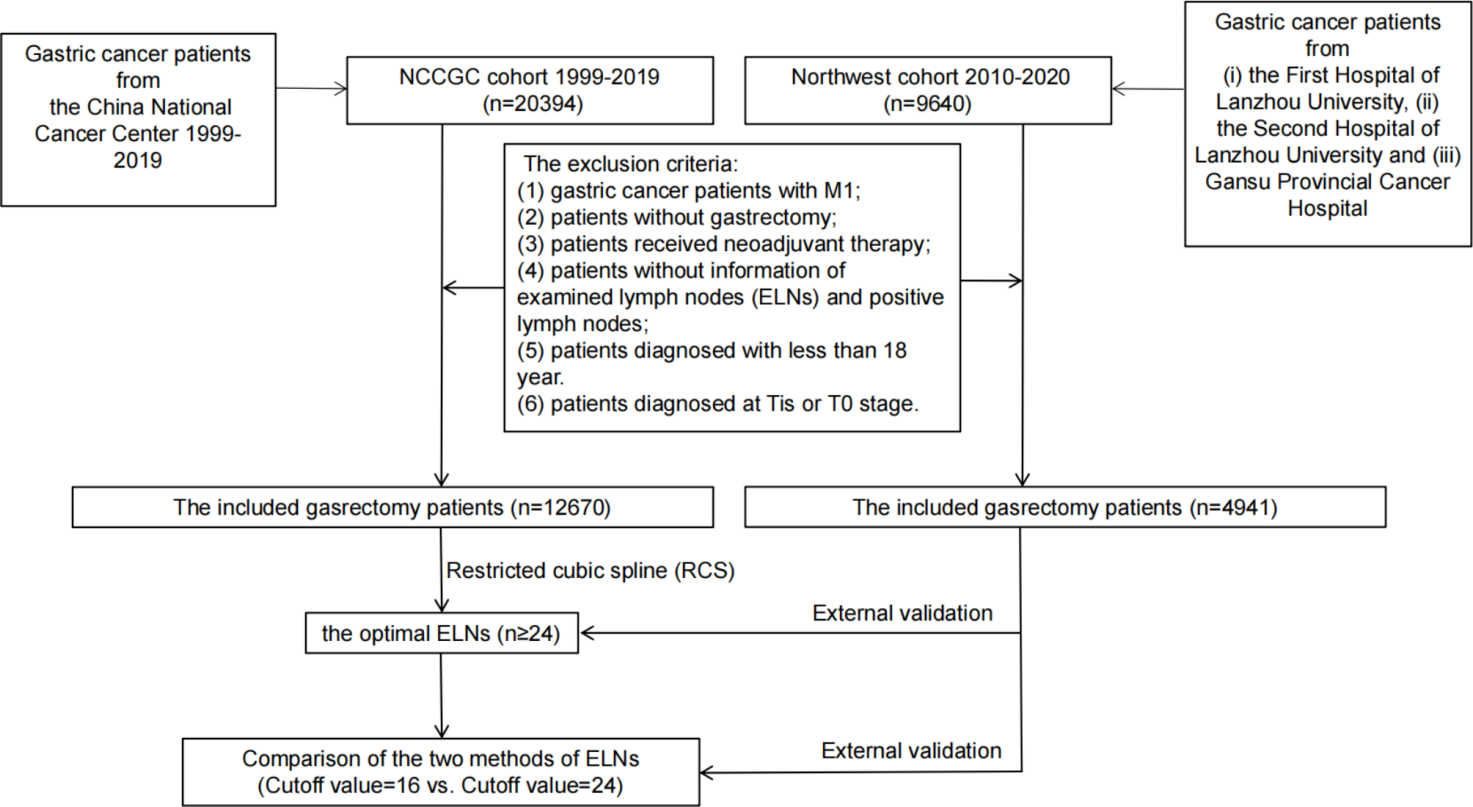
Flow diagram illustrating recruitment of gastrectomy patients

In this cohort study, NCCGC data from 20,394 consecutive gastric cancer patients who underwent gastrectomy between 1999 and 2019 were retrospectively reviewed. The geographical locations of these 20,394 patients of NCCGC cohort showed in Fig. 1A. The exclusion criteria were as follows: [1] gastric cancer patients with M1; [2] no gastrectomy; [3] patients received neoadjuvant therapy; [4] patients without information of ELNs and positive lymph nodes; [5] patients diagnosed with less than 18 years; and [6] patients diagnosed at Tis or T0 stage. After selecting the data, 12,670 gastrectomy patients were included in this study (Fig. 2).

Moreover, we included an additional 4941 gastric cancer patients from a Northwest cohort 2010–2020 year as external verification based on the same exclusion criteria. These gastric cancer patients all came from northwest China (Shanxi, Gansu, Qinghai, Xinjiang and Ningxia province, Fig. 1B) at 3 institutions (the First Hospital of Lanzhou University, Lanzhou University Second Hospital, and Gansu Provincial Cancer Hospital).

### The optimal threshold for ELNs and validation

We used RCS models fitted for Cox proportional hazards models with 4 knots to explore the optimal threshold for ELNs after adjusting sex, age at diagnosis, loss weight, body mass index (BMI), tumor location, pathological T stage, pathological N stage, grade, vascular invasion, nerve invasion, and adjuvant therapy. The RCS method was a popular way to flexibly model non-linear relationships in regression models [12]. Here, we conducted the RCS model to examine relationships between ELNs and survival of gastric cancer patients. Comparisons between the groups (ELN < 24 and ELN ≥ 24) were evaluated using a t-test for continuous variables and a chi-square test for categorical variables. Overall survival (OS) was calculated using the Kaplan-Meier method, and differences between the survival curves were assessed using the log-rank test. Univariate and multivariate Cox proportional hazards models were used to identify the prognostic factors. Variables with a P value of less than 0.10 in the univariate analysis were included in the multivariate analysis. The hazard ratio (HR) and 95% confidence interval (CI) were used to measure the risk of death. Next, we determined the optimal threshold for ELNs in the Northwest cohort using univariate and multivariate analysis.

### Statistical analysis

All statistical analyses were done using R (version 4.1.0) and SPSS (version 26). A p-value of less than 0.05 was considered to be statistically significant and all the tests were two-sided.

### Role of the funding source

This work was supported by the grant from National Key R&D Program of China (No. 2017YFC0908300) and Non-profit Central Research Institute Fund of Chinese Academy of Medical Sciences (No. 2021-RC310-009).

## Results

**Fig. 3:**
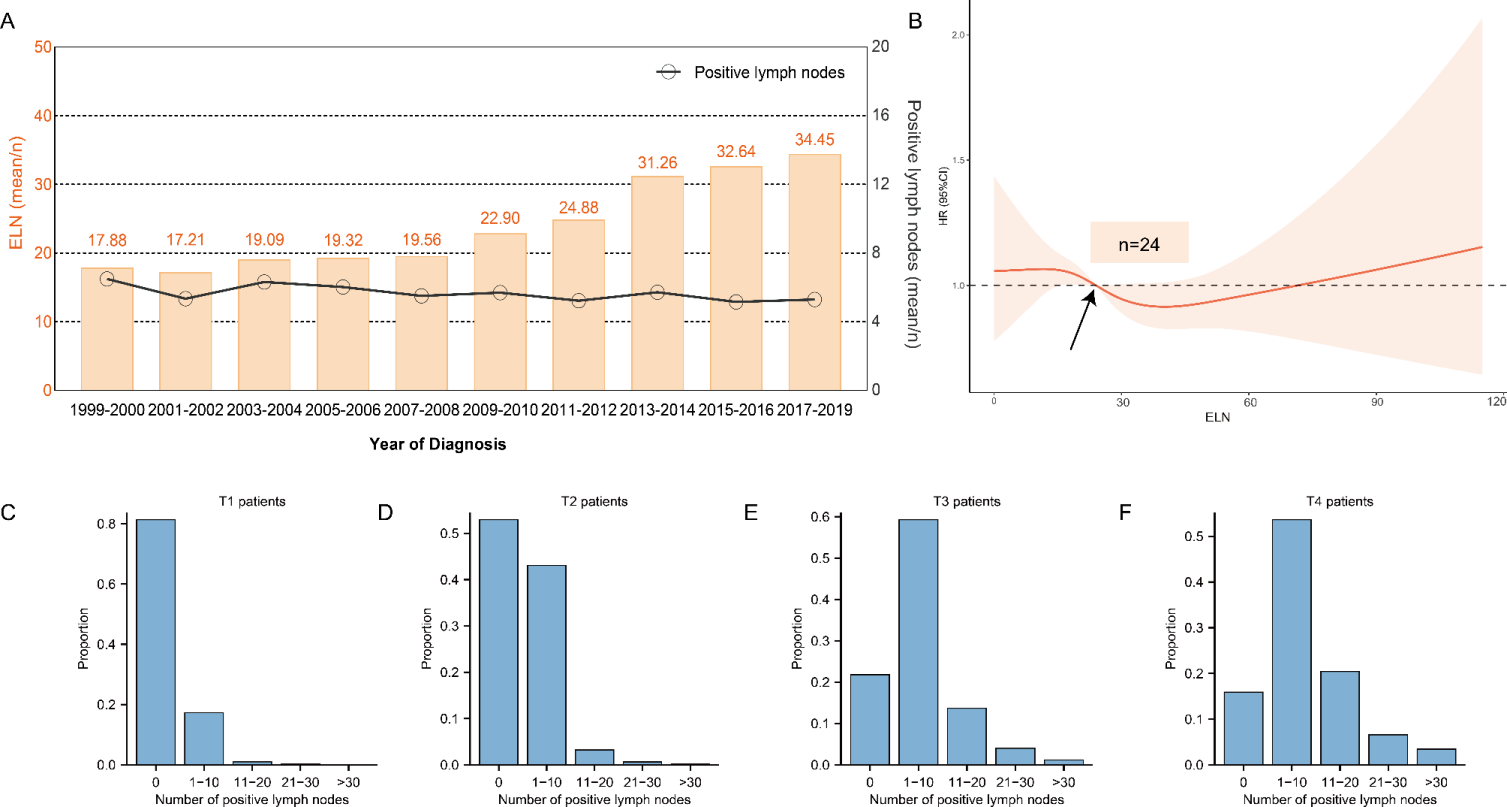
(**A**) Time-trend of the number of positive lymph nodes and ELNs in NCCGC cohort 1999–2019 year, (**B**) the optimal threshold of ELNs was 24 (HR = 1.00) from RCS model, (**C-F**) the proportion of different positive lymph node groups (0, 1–10, 11–20, 21–30, > 30 nodes) by pathological T stage subgroup

In this study, 12,670 gastrectomy patients were included in the NCCGC cohort and 4941 patients in the Northwest cohort. We sought to characterize time-trend of the number of positive lymph nodes and ELNs in NCCGC cohort 1999–2019 year (Fig. 3A). During this period, the average number of ELNs increased from 17.88 to 34.45 nodes, while the number of positive lymph nodes remained stable (5–6 nodes). The proportion of different positive lymph node groups (0, 1–10, 11–20, 21–30, > 30 nodes) by pathological T stage subgroup are represented in Fig. 3C-F. The proportion of positive lymph nodes of gastrectomy patients were 18.69% in T1 stage; however, the proportion rose rapidly with 47.06% in T2 patients, 78.18% in T3 patients, and 84.11% in T4 patients.

To examine the relationship between the number of ELNs and survival of gastric cancer patients, we conducted Cox proportional regression analyses using the RCS method. In Fig. 3B, the RCS model showed a U-curved association between ELNs and the risk of all-cause mortality, and the optimal threshold of ELNs was 24 (HR = 1.00).

Thus, the clinicopathological features of gastrectomy patients with different ELN groups (ELN < 24 vs. ELN ≥ 24) were compared in both NCCGC cohort and Northwest cohort (Table 1). Compare to patients with ELN < 24 in NCCGC cohort, the ELN ≥ 24 group showed higher proportion of female (25.0% vs. 27.4%, p = 0.002), diffuse type (32.4% vs. 39.2%, p < 0.001), pathological T3 (22.0% vs. 26.3%, p < 0.001), pathological N3 (21.7% vs. 36.9%, p < 0.001), poorly differentiation (46.8% vs. 53.4%, p < 0.001), nerve invasion (19.3% vs. 39.7%) and vascular invasion (29.1% vs. 42.5%, p < 0.001). In addition, relatively higher percentages of proximal location (45.5% vs. 31.2%, p < 0.001) and pathological N0 (37.5% vs. 32.2%, p < 0.001) were shown in ELN < 24 group as compared to ELN ≥ 24 group.

**Table 1:** Characteristics of gastrectomy patients from NCCGC cohort and Northwest cohort

Characteristics		NCCGC cohort (N = 12,670)		P value	Northwest cohort (n = 4941)		P value
		ELN < 24 (N = 6176)	ELN ≥ 24 (N = 6494)		ELN < 24 (N = 2732)	ELN ≥ 24 (N = 2209)	
		N (%)	N (%)		N (%)	N (%)	
Age at diagnosis (years)							
	18–34	171 (2.8)	173 (2.7)	< 0.001	38 (1.4)	48 (2.2)	0.001
	35–50	1164 (19.0)	1233 (19.1)		454 (16.6)	406 (18.4)	
	51–64	2670 (43.5)	3090 (47.8)		1275 (46.7)	1079 (48.8)	
	≥ 65	2126 (34.7)	1967 (30.4)		965 (35.3)	676 (30.6)	
Gender							
	Male	4631 (75.0)	4709 (72.6)	0.002	2045 (74.9)	1687 (76.4)	0.218
	Female	1541 (25.0)	1774 (27.4)		687 (25.1)	522 (23.6)	
Smoking status							
	Smokers	2424 (40.0)	2892 (45.6)	< 0.001	393 (14.5)	436 (19.8)	< 0.001
	Never smokers	3642 (60.0)	3453 (54.4)		2323 (85.5)	1763 (80.2)	
Alcohol consumption							
	Drinkers	1903 (31.4)	2480 (39.1)	< 0.001	235 (8.7)	271 (12.3)	< 0.001
	Never drinkers	4159 (68.6)	3859 (60.9)		2481 (91.3)	1928 (87.7)	
Location							
	Proximal	2766 (45.5)	2015 (31.2)	< 0.001	464 (18.2)	432 (20.1)	0.214
	Distal	3184 (52.4)	4136 (64.1)		1904 (74.7)	1575 (73.3)	
	Total	125 (2.1)	303 (4.7)		182 (7.1)	141 (6.6)	
Lauren type							
	Intestinal	958 (42.6)	1563 (34.2)	< 0.001	505 (34.8)	285 (26.0)	< 0.001
	Diffuse	729 (32.4)	1792 (39.2)		558 (38.5)	426 (38.8)	
	Mixed	563 (25.0)	1220 (26.7)		387 (26.7)	387 (35.2)	
BMI (kg/m2) at diagnosis							
	< 18.5	341 (5.6)	332 (5.2)	0.032	187 (9.1)	290 (14.4)	< 0.001
	18.5–24	2900 (47.9)	3198 (50.4)		1315 (63.8)	1285 (63.8)	
	24–28	2142 (35.4)	2170 (34.2)		467 (22.7)	370 (18.4)	
	>28	671 (11.1)	642 (10.1)		92 (4.5)	69 (3.4)	
Weight loss (kg)							
	0–2	3603 (71.1)	4281 (72.6)	0.178	1564 (62.1)	1062 (53.8)	< 0.001
	2.0–5	1011 (19.9)	1088 (18.4)		669 (26.6)	652 (33.0)	
	5–10	364 (7.2)	438 (7.4)		232 (9.2)	214 (10.8)	
	> 10	90 (1.8)	93 (1.6)		54 (2.1)	45 (2.3)	
Surgical Margin							
	Negative	5811 (96.6)	6271 (97.9)	< 0.001	2556 (96.9)	2103 (98.1)	0.011
	Positive	207 (3.4)	135 (2.1)		81 (3.1)	41 (1.9)	
Surgical approach							
	Open	5544 (91.9)	4697 (73.2)	< 0.001	1051 (40.9)	1154 (54.1)	< 0.001
	Laparoscopy-assisted	376 (6.2)	1290 (20.1)		1042 (40.6)	663 (31.1)	
	Conversion to laparotomy	18 (0.3)	86 (1.3)		71 (2.8)	57 (2.7)	
	Total laparoscopy	95 (1.6)	342 (5.3)		405 (15.8)	260 (12.2)	
Pathologic T stage							
	T1	1255 (20.7)	1255 (19.5)	< 0.001	407 (17.1)	273 (13.8)	< 0.001
	T2	668 (11.0)	762 (11.8)		329 (13.8)	224 (11.4)	
	T3	1335 (22.0)	1690 (26.3)		484 (20.3)	480 (24.3)	
	T4	2797 (46.2)	2729 (42.4)		1167 (48.9)	996 (50.5)	
Pathologic N stage							
	N0	2279 (37.5)	2075 (32.2)	< 0.001	1014 (42.0)	638 (32.2)	< 0.001
	N1	1175 (19.4)	901 (14.0)		483 (20.0)	386 (19.5)	
	N2	1300 (21.4)	1093 (17.0)		439 (18.2)	356 (18.0)	
	N3	1317 (21.7)	2375 (36.9)		479 (19.8)	601 (30.3)	
Grade							
	Poorly	2735 (46.8)	3374 (53.4)	< 0.001	814 (33.0)	867 (41.7)	< 0.001
	Poorly-Moderately	1438 (24.6)	1625 (25.7)		759 (30.7)	696 (33.5)	
	Moderately	1297 (22.2)	1084 (17.2)		657 (26.6)	394 (18.9)	
	Well-Moderately	187 (3.2)	109 (1.7)		133 (5.4)	80 (3.8)	
	Well	190 (3.2)	120 (1.9)		98 (4.0)	42 (2.0)	
	Undifferentiated	2 (0.0)	1 (0.0)		8 (0.3)	1 (0.0)	
Signet ring cell							
	Yes	1637 (26.8)	2160 (33.5)	< 0.001	461 (22.7)	392 (21.3)	0.284
	No	4461 (73.2)	4294 (66.5)		1571 (77.3)	1452 (78.7)	
Nerve invasion							
	Yes	1171 (19.3)	2559 (39.7)	< 0.001	1105 (53.5)	876 (54.5)	0.543
	No	4894 (80.7)	3891 (60.3)		959 (46.5)	730 (45.5)	
Vascular invasion							
	Yes	1799 (29.1)	2760 (42.5)	< 0.001	1514 (55.4)	1179 (53.4)	0.151
	No	4377 (70.9)	3734 (57.5)		1218 (44.6)	1030 (46.6)	
Linitis plastica							
	No	6051 (99.7)	6393 (99.4)	0.076	1580 (97.9)	1616 (98.4)	0.330
	Yes	21 (0.3)	36 (0.6)		34 (2.1)	27 (1.6)	
Adjuvant therapy							
	Yes	2124 (76.7)	2575 (79.7)	0.006	1739 (71.5)	1544 (74.0)	0.066
	No	644 (23.3)	657 (20.3)		692 (28.5)	543 (26.0)	

**Fig. 4:**
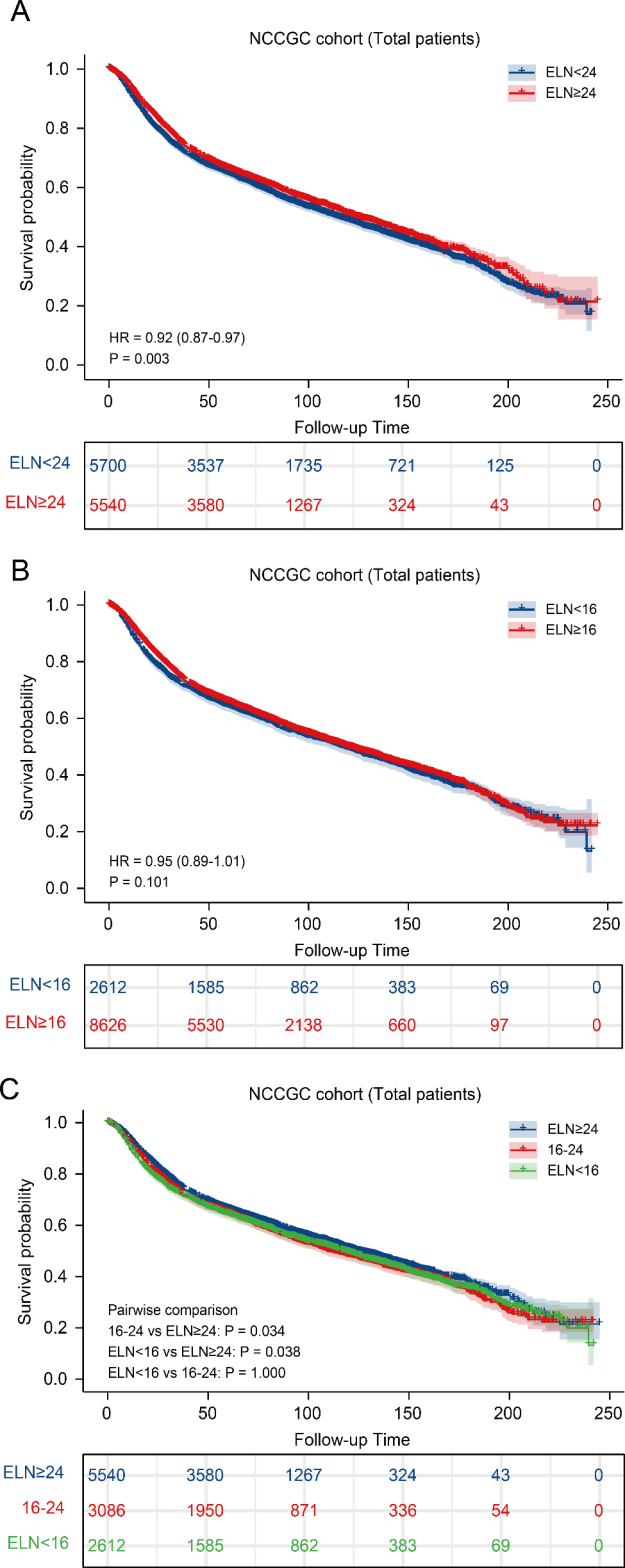
The survival results by number of ELNs are depicted by Kaplan-Meier curves in (**A**) ELN < 24 and ELN ≥ 24; (**B**) ELN < 16 and ELN ≥ 16, and (**C**) ELN < 16, 16–23 and ELN ≥ 24.

The survival results by number of ELNs are depicted in Fig. 4 by Kaplan-Meier curves. The ELN ≥ 24 group had a better OS than the ELN < 24 group clearly (Fig. 4A, P = 0.003). With respect to the threshold of 16 ELNs, there was no significantly difference between the two groups (Fig. 4B, P = 0.101). In order to explore which threshold (cutoff = 16 vs. cutoff = 24) predicts prognosis better, we divided the NCCGC cohort patients divided into 3 groups (ELN < 16, 16–23 and ≥ 24), and found that the OS of ELN ≥ 24 patients was significantly higher than both ELN < 16 and 16–23 groups (Fig. 4C, P = 0.038 and 0.034, respectively). However, no difference in survival outcomes was also observed between ELN < 16 and 16–23 groups (P = 1.00).

**Fig. 5:**
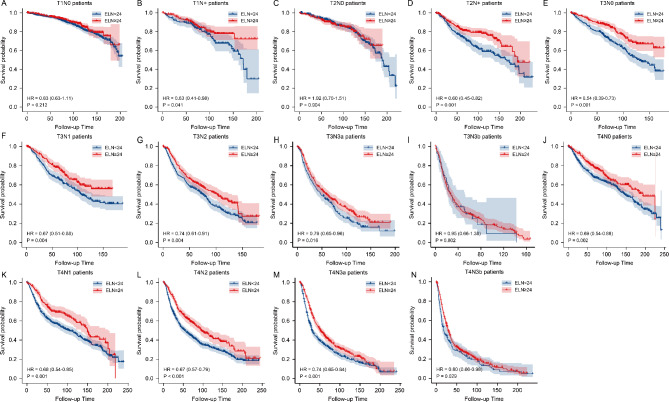
The Kaplan-Meier survival curves of gastric cancer patients between ELN < 24 and ELN ≥ 24 groups in T1N0, T1N+, T2N0, T2N+, T3N0, T3N1, T3N2, T3N3a, T3N3b, T4N0, T4N1, T4N2, T4N3a and T4N3b.

**Fig. 6:**
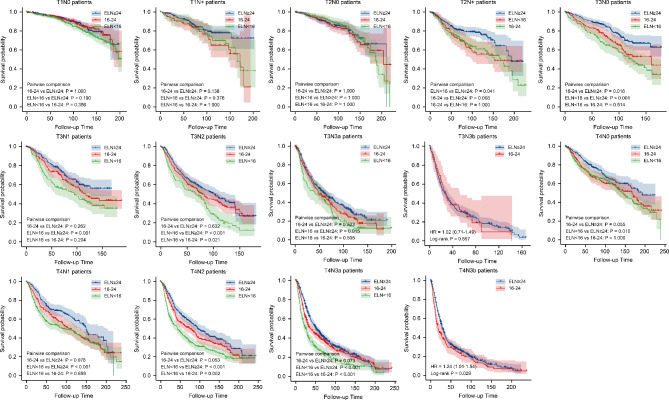
The Kaplan-Meier survival analysis among ELN < 16, 16–23 and ELN ≥ 24 groups in T1N0, T1N+, T2N0, T2N+, T3N0, T3N1, T3N2, T3N3a, T3N3b, T4N0, T4N1, T4N2, T4N3a and T4N3b.

Next, we conducted subgroup analysis based on pathological T and pathological N stage We further finished the subgroup survival analysis with different ELNs groups by pathological T and N stage. Figure 5 showed the Kaplan-Meier survival curves of gastric cancer patients between ELN < 24 and ELN ≥ 24 groups, and we found obvious survival benefit of ELN ≥ 24 group in T1N+ (P = 0.041), T2N+ (P = 0.001), T3N0 (P < 0.001), T3N1 (P = 0.004), T3N2 (P = 0.004), T3N3a (P = 0.016), T4N0 (P = 0.002), T4N1(P = 0.001), T4N2(P < 0.001), T4N3a stage (P < 0.001) and T4N3b stage (P = 0.029). We also performed detailed Kaplan-Meier survival analysis among ELN < 16, 16–23 and ELN ≥ 24 groups by pathological T and pathological N stage subgroup (Fig. 6), and found obvious survival benefits of ELN ≥ 24 patients than those with ELN < 16 and 16–23.

The univariate and multivariate Cox proportional hazards models were used to determine the prognostic factors for OS with different ELN groups in NCCGC cohort (Supplementary Table 1, Supplementary Table 2, and Table 2). After adjusting for age at diagnosis, gender, smoking status, alcohol consumption, tumor location, BMI, weight loss, surgical Margin, pathological T stage, pathological N stage, grade, signet ring cell, nerve invasion, vascular invasion, linitis plastica and adjuvant therapy, the multivariate analysis found survival benefits in total gastrectomy patients with ELN ≥ 24 (HR = 0.787, 95%CI: 0.711–0.870, P < 0.001), as well as the subgroups of T2 patients (HHR = 0.621, 95%CI: 0.399–0.966, P = 0.035), T3 patients (HR = 0.787, 95%CI: 0.659–0.940, P = 0.008) and T4 patients (HR = 0.775, 95%CI: 0.675–0.888, P < 0.001). As for the cutoff value of 16 ELNs, multivariate analysis revealed that ELN ≥ 16 group was also associated with better survival outcomes of gastrectomy patients (HR = 0.733, 95%CI: 0.647–0.831, P < 0.001). However, after stratification by pathological T stage, the survival advantage was only found in T3 patients (HR = 0.710, 95%CI: 0.558–0.902, P = 0.005) and T4 patients (HR = 0.773, 95%CI: 0.671–0.891, P < 0.001).

**Table 2:** The multivariate analysis of gastrectomy patients with different ELN groups (Cutoff = 24 vs. Cutoff = 16) in NCCGC cohort

Characteristics	NCCGC cohort (Cutoff = 24)			NCCGC cohort (Cutoff = 16)			
	HR	95%CI	P value		HR	95%CI	P value
Total patients							
ELN < 24	1			ELN < 16	1		
ELN ≥ 24	0.787	0.711–0.870	< 0.001	ELN ≥ 16	0.733	0.647–0.831	< 0.001
T1 patients							
ELN < 24	1			ELN < 16	1		
ELN ≥ 24	1.043	0.618–1.763	0.874	ELN ≥ 16	1.202	0.691–2.091	0.514
T2 patients							
ELN < 24	1			ELN < 16	1		
ELN ≥ 24	0.621	0.399–0.966	0.035	ELN ≥ 16	0.812	0.476–1.385	0.444
T3 patients							
ELN < 24	1			ELN < 16	1		
ELN ≥ 24	0.787	0.659–0.940	0.008	ELN ≥ 16	0.710	0.558–0.902	0.005
T4 patients							
ELN < 24	1			ELN < 16	1		
ELN ≥ 24	0.775	0.675–0.888	< 0.001	ELN ≥ 16	0.773	0.671–0.891	< 0.001

*Adjusted for age at diagnosis, gender, smoking status, alcohol consumption, tumor location, BMI, weight loss, surgical Margin, pathologic T stage, pathologic N stage, grade, signet ring cell, nerve invasion, vascular invasion, linitis plastica and adjuvant therapy

We compared the prognosis among the three different ELNs groups with ELN < 16, 16–23 and ELN ≥ 24 (Table 3). Compared to patients with ELN ≥ 24, patients with ELN < 16 and 16–23 conveyed worse survival outcomes (HR = 1.461, 95%CI: 1.279–1.670, P < 0.001; and HR = 1.190, 95%CI: 1.062–1.335, P = 0.003; respectively). The survival differences also appeared in T2-4 stage subgroup, suggesting patients with ELN ≥ 24 was a prognostic factor associated with favorable survival outcomes in T2 patients (HR = 1.762, 95%CI: 1.141–2.720, P = 0.011; and HR = 1.573, 95%CI: 1.045–2.368, P = 0.030; respectively), T3 patients (HR = 1.431, 95%CI: 1.118–1.832, P = 0.004; and HR = 1.181, 95%CI: 0.969–1.437, P = 0.099; respectively) and T4 patients (HR = 1.478, 95%CI: 1.240–1.761, P < 0.001; and HR = 1.179, 95%CI: 1.007–1.379, P = 0.040; respectively).

**Table 3:** The multivariate analysis of gastrectomy patients with different ELN groups (ELN < 16, 16–23, and ≥ 24) in NCCGC cohort

Characteristics		NCCGC cohort		
		HR	95%CI	P value
Total patients				
	ELN ≥ 24	1		
	ELN < 16	1.461	1.279–1.670	< 0.001
	ELN 16–23	1.190	1.062–1.335	0.003
T1 patients				
	ELN ≥ 24	1		
	ELN < 16	0.922	0.492–1.727	0.799
	ELN 16–23	1.173	0.659–1.086	0.588
T2 patients				
	ELN ≥ 24	1		
	ELN < 16	1.762	1.141–2.720	0.011
	ELN 16–23	1.573	1.045–2.368	0.030
T3 patients				
	ELN ≥ 24	1		
	ELN < 16	1.431	1.118–1.832	0.004
	ELN 16–23	1.181	0.969–1.437	0.099
T4 patients				
	ELN ≥ 24	1		
	ELN < 16	1.478	1.240–1.761	< 0.001
	ELN 16–23	1.179	1.007–1.379	0.040

*Adjusted for age at diagnosis, gender, smoking status, alcohol consumption, tumor location, BMI, weight loss, surgical Margin, pathologic T stage, pathologic N stage, grade, signet ring cell, nerve invasion, vascular invasion, linitis plastica and adjuvant therapy

In order to verify the optimal threshold of ELNs (Cutoff = 24), we performed univariate and multivariate analysis in the Northwest cohort for external validation. As showed in Supplementary Table 3, ELN ≥ 24 group was associated with improved OS in multivariate analysis of gastric cancer patients (HR = 0.754, 95%CI: 0.610–0.932, P = 0.009). Moreover, gastrectomy patients with ELN < 16 (HR = 1.460, 95%CI: 1.068–1.996, P = 0.018) and 16–23 (HR = 1.275, 95%CI: 1.010–1.611, P = 0.041) were significantly worse than those with ELN ≥ 24 in multivariate analysis (Table 4).

**Table 4:** The univariate and multivariate analysis of gastrectomy patients with different ELN groups (ELN < 16, 16–23, and ≥ 24) in Northwest cohort

Characteristics	Univariate analysis			Multivariate analysis		
	HR	95%CI	P value	HR	95%CI	P value
ELN						
≥ 24	1			1		
< 16	0.997	0.881–1.129	0.967	1.460	1.068–1.996	0.018
16–24	1.077	0.881–1.129	0.186	1.275	1.010–1.611	0.041

*Adjusted for age at diagnosis, gender, smoking status, alcohol consumption, tumor location, BMI, weight loss, surgical Margin, pathologic T stage, pathologic N stage, grade, signet ring cell, nerve invasion, vascular invasion, linitis plastica and adjuvant therapy

## Discussion

This multicenter study investigated systematically the minimum number of ELNs that should be retrieved during gastrectomy for optimal survival using RCS model and verified based on a cohort from the highest gastric cancer incidence areas. To the best of our knowledge, our analysis represents the largest evaluation of optimal threshold of ELNs for gastric cancer patients without neoadjuvant therapy in China, with the number of 12,670 patients in NCCGC cohort and 4941 patients in Northwest cohort. There were two main findings in this study: (i) ELN ≥ 24 maintained excellent prognostic value in total gastrectomy patients, as well as subgroup analysis in pathological T2-4 gastric cancer patients; and (ii) there was no statistical difference between ELN < 16 and ELN ≥ 16 groups for gastric cancer patients with pathological T2 in multivariate analysis, indicating that the ELNs number of 16 was not enough to achieve survival benefits.

In this study, we observed an obvious upward trend of average number of ELNs during 1999–2019 year in NCCGC cohort, with an increase of almost double from 17.88 to 34.45 nodes. The similar improved trend was also observed in the SEER database [13]. A mainly reason was that the clinical significance of extended lymphadenectomy as well as more ELNs of gastrectomy patients had been evaluated in many studies [14, 15]. Secondly, emerging surgical procedures like laparoscopic surgery, as well as standardized D2/D2 + procedures, also had played an important role for the increasing nodes retrieved in gastric cancer [16, 17]. Encouragingly, the huge number of gastric cancer patients with large number of lymph nodes retrieved in NCCGC cohort provided data support for exploring minimum number of ELNs for optimal survival.

According to the 8th Edition of the American Joint Committee on Cancer (AJCC) TNM Staging System, it is recommended that ELN ≥ 16 could be removed for accurate staging, which allows for distinction of N3a (7–15 nodes) and N3b (≥ 16 nodes) disease due to the importance of N status to final TNM stage [2]. However, increasing evidence demonstrated that it is not sufficient for gastric cancer patients with ≥ 16 ELNs in terms for a better prognosis, particularly for pathological N3b stage patients [11, 18, 19]. In our study, the average of positive lymph nodes increased with increasing pathological T stage, and up to 85% in T4 patients, which was similar with previous studies. Specifically, more than 18% T4 patients with ≥ 16 positive lymph nodes, showing the high node burden for gastrectomy patients. In this context, we compared the ELN < 16 and ELN ≥ 16 patients in univariate and multivariate analysis. The results revealed that ELN ≥ 16 was a protective factor for total gastrectomy patients in NCCGC cohort, however, no significant difference was observed in subgroup of T2 patients. Taken together, the threshold of 16 ELNs was insufficient for achieving the survival benefits for gastrectomy patients with pathological T2.

Undoubtedly, higher ELNs was associated with prolonged survival in patients with gastric cancer regardless of the neoadjuvant therapy status [6, 7, 9, 11, 13, 20, 21]. Smith et al. [6] have demonstrated that the greater number of ELNs, the better was the resulting survival of gastric cancer patients using 3814 patients from SEER database. Specifically, each increase in 10 ELNs resulted in an approximately 7% increase in survival for gastrectomy patients. In this context, we assumed that there is an optimal minimal number should be definable for patients with gastric cancer. RCS model, a powerful tool in the analysis of non-linear associations between continuous variables and outcome [22, 23], provides new insights to confirm the best cutoff value for ELNs. Here, we defined the optimal threshold of ELNs was 24 based on the NCCGC cohort using RCS models, and found that ELN ≥ 24 group showed clear separation from patients with ELN < 24 of Kaplan-Meier curves in T1N+, T2N+, T3N0, T3N1, T3N2, T3N3a, T4N0, T4N1, T4N2, T4N3a and T4N3b stage (P < 0.005). We observed that the survival benefit did not occur in T3N3b patients, mainly because the sample size was too small for these patients. As for multivariate analysis, ELN ≥ 24 group presented as an independent predictor for better survival in total gastric cancer patients, as well as subgroup analysis in pathological T2, T3 and T4 patients.

What we further need to consider is that whether the number of 24 could replace 16 as the optimal minimal threshold of ELNs for gastrectomy patients. In our study of multivariate analysis, ELN ≥ 24 group was significantly related to the OS improvement of pathological T2, T3 and T4 patients, but this survival advantage of ELN ≥ 16 group was only reflected in T3 and T4 patients, suggesting ELN ≥ 24 was a more universal cutoff value for gastric cancer patients. In addition, ELN ≥ 24 also showed improved survival than both ELN < 16 and 16–23 groups not only in NCCGC cohort but also in the Northwest cohort. Recently, Adrienne et al. showed that the patients received neoadjuvant therapy with at least 23 nodes demonstrated an improved 5-year OS [9]. Siewert et al. observed that resection of 25 nodes was associated with increased 10-year survival for patients with stage II disease [10]. Therefore, we believe that the wide application of ELN ≥ 24 would provide a firm foundation for personalized treatment of gastric cancer in the future.

This study had some limitations. Firstly, both and NCCGC cohort and Northwest cohort were retrospective cohorts, which may have some inherent biases and unknown confounders. Secondly, the true representativeness of our study to the actual Chinese population can be debated given that the NCCGC cohort does not provide population-level data. Thirdly, data on surgical complications is not available. Fourthly, data were used that only included patients who did not undergo neoadjuvant therapy because of its potential to affect pathological staging of the resected specimens. The optimal number of ELNs for patients received neoadjuvant therapy are also be considered in the future clinical practice. Despite all this, this larger study conducted and tested the evaluation of optimal threshold of ELNs for gastric cancer patients without neoadjuvant therapy in China, which would play an important role in guiding the number of ELNs in the future.

In conclusion, the minimum number of ELNs for optimal survival of gastric cancer with pathological T2-4 was 24.

### Electronic supplementary material

Below is the link to the electronic supplementary material.

Supplementary Material 1

## Data Availability

The data from NCCGC cohort and Northwest cohort used to support this finding of this study is include in tables within the article.
