# Supplementary material for: The minimum number of examined lymph nodes was 24 for optimal survival of pathological T2-4 gastric cancer: a multi-center, hospital-based study covering 20 years of data
Source: BMC Cancer. 2023 Sep 21;23:892. doi: 10.1186/s12885-023-11138-0 (PMC10512540; doi:10.1186/s12885-023-11138-0)
Supplement: Supplementary file 1 — Supplementary Material 1 [file 12885_2023_11138_MOESM1_ESM.docx]

Supplementary Table 1. The univariate analysis of gastrectomy patients with different ELN groups (ELN <24 and ≥24) in NCCGC cohort.

| Characteristics | | NCCGC cohort | | | T1 patients | | | T2 patients | | | T3 patients | | | T4 patients | | |
| --- | --- | --- | --- | --- | --- | --- | --- | --- | --- | --- | --- | --- | --- | --- | --- | --- |
|  |  | HR | 95%CI | P value | HR | 95%CI | P value | HR | 95%CI | P value | HR | 95%CI | P value | HR | 95%CI | P value |
| ELN | |  |  |  |  |  |  |  |  |  |  |  |  |  |  |  |
|  | <24 | 1 |  |  | 1 |  |  | 1 |  |  | 1 |  |  | 1 |  |  |
|  | ≥24 | 0.937 | 0.887-0.990 | 0.021 | 0.813 | 0.640-1.031 | 0.088 | 0.770 | 0.608-0.975 | 0.030 | 0.898 | 0.810-0.996 | 0.042 | 0.954 | 0.888-1.025 | 0.201 |
| Pathologic T stage | |  |  |  |  |  |  |  |  |  |  |  |  |  |  |  |
|  | T1 | 1 |  |  | - |  |  | - |  |  | - |  |  | - |  |  |
|  | T2 | 1.758 | 1.502-2.059 | <0.001 |  |  |  |  |  |  |  |  |  |  |  |  |
|  | T3 | 5.033 | 4.455-5.687 | <0.001 |  |  |  |  |  |  |  |  |  |  |  |  |
|  | T4 | 6.157 | 5.481-6.916 | <0.001 |  |  |  |  |  |  |  |  |  |  |  |  |
| Age at diagnosis (years) | |  |  |  |  |  |  |  |  |  |  |  |  |  |  |  |
|  | 18-34 | 1 |  |  | 1 |  |  | 1 |  |  | 1 |  |  | 1 |  |  |
|  | 35-50 | 1.048 | 0.855-1.284 | 0.651 | 2.225 | 1.440-3.438 | <0.001 | 1.164 | 0.455-2.977 | 0.751 | 0.882 | 0.562-1.383 | 0.584 | 0.925 | 0.730-1.173 | 0.522 |
|  | 51-64 | 1.156 | 0.950-1.406 | 0.147 | 7.075 | 4.647-10.773 | <0.001 | 1.564 | 0.639-3.827 | 0.328 | 0.970 | 0.628-1.498 | 0.889 | 0.861 | 0.685-1.083 | 0.201 |
|  | ≥65 | 1.803 | 1.482-2.194 | <0.001 | 0.240 | 0.033-1.773 | 0.162 | 2.960 | 1.213-7.222 | 0.017 | 1.329 | 0.860-2.054 | 0.200 | 1.174 | 0.933-1.476 | 0.171 |
| Gender | |  |  |  |  |  |  |  |  |  |  |  |  |  |  |  |
|  | Male | 1 |  |  | 1 |  |  | 1 |  |  | 1 |  |  | 1 |  |  |
|  | Female | 0.895 | 0.840-0.954 | 0.001 | 0.586 | 0.444-0.773 | <0.001 | 0.748 | 0.563-0.994 | 0.046 | 1.058 | 0.933-1.200 | 0.381 | 0.963 | 0.888-1.044 | 0.355 |
| Smoking status | |  |  |  |  |  |  |  |  |  |  |  |  |  |  |  |
|  | Smokers | 1 |  |  | 1 |  |  | 1 |  |  | 1 |  |  | 1 |  |  |
|  | Never smokers | 0.909 | 0.859-0.961 | 0.001 | 0.788 | 0.624-0.995 | 0.045 | 0.919 | 0.728-1.160 | 0.478 | 0.981 | 0.884-1.088 | 0.716 | 0.950 | 0.883-1.022 | 0.168 |
| Alcohol consumption | |  |  |  |  |  |  |  |  |  |  |  |  |  |  |  |
|  | Drinkers | 1 |  |  | 1 |  |  | 1 |  |  | 1 |  |  | 1 |  |  |
|  | Never drinkers | 0.959 | 0.905-1.017 | 0.160 | 0.994 | 0.772-1.281 | 0.963 | 0.877 | 0.687-1.120 | 0.293 | 1.019 | 0.915-1.135 | 0.726 | 0.977 | 0.906-1.055 | 0.555 |
| Location | |  |  |  |  |  |  |  |  |  |  |  |  |  |  |  |
|  | Proximal | 1 |  |  | 1 |  |  | 1 |  |  | 1 |  |  | 1 |  |  |
|  | Distal | 0.657 | 0.621-0.694 | <0.001 | 0.508 | 0.399-0.646 | <0.001 | 0.619 | 0.490-0.782 | <0.001 | 0.781 | 0.701-0.869 | <0.001 | 0.889 | 0.826-0.956 | 0.002 |
|  | Total | 1.020 | 0.866-1.201 | 0.813 | 0.751 | 0.329-1.713 | 0.496 | 0.628 | 0.307-1.285 | 0.203 | 1.045 | 0.761-1.436 | 0.783 | 1.250 | 1.018-1.535 | 0.033 |
| BMI (kg/m2) at diagnosis | |  |  |  |  |  |  |  |  |  |  |  |  |  |  |  |
|  | <18.5 | 1 |  |  | 1 |  |  | 1 |  |  | 1 |  |  | 1 |  |  |
|  | 18.5-24 | 0.834 | 0.744-0.936 | 0.002 | 1.345 | 0.747-2.423 | 0.323 | 0.457 | 0.302-0.691 | <0.001 | 0.684 | 0.952-0.750 | 1.207 | 0.872 | 0.755-1.006 | 0.061 |
|  | 24-28 | 0.713 | 0.634-0.803 | <0.001 | 1.302 | 0.717-2.363 | 0.386 | 0.391 | 0.255-0.601 | <0.001 | 0.796 | 0.623-1.015 | 0.066 | 0.793 | 0.683-0.920 | 0.002 |
|  | ＞28 | 0.657 | 0.571-0.756 | <0.001 | 0.898 | 0.453-1.781 | 0.759 | 0.320 | 0.193-0.530 | <0.001 | 0.959 | 0.729-1.260 | 0.762 | 0.738 | 0.614-0.886 | 0.001 |
| Weight loss (kg) | |  |  |  |  |  |  |  |  |  |  |  |  |  |  |  |
|  | 0-2 | 1 |  |  | 1 |  |  | 1 |  |  | 1 |  |  | 1 |  |  |
|  | 2.0-5 | 1.413 | 1.314-1.520 | <0.001 | 1.621 | 1.180-2.226 | 0.003 | 0.982 | 0.702-1.374 | 0.916 | 1.225 | 1.070-1.403 | 0.003 | 1.193 | 1.085-1.312 | <0.001 |
|  | 5-10 | 1.724 | 1.556-1.909 | <0.001 | 1.228 | 0.683-2.206 | 0.493 | 1.614 | 0.968-2.693 | 0.066 | 1.425 | 1.163-1.747 | 0.001 | 1.318 | 1.161-1.496 | <0.001 |
|  | >10 | 1.915 | 1.573-2.332 | <0.001 | 4.888 | 2.009-11.895 | <0.001 | 0.785 | 0.194-3.179 | 0.734 | 1.643 | 1.066-2.533 | 0.024 | 1.361 | 1.073-1.724 | 0.011 |
| Surgical Margin | |  |  |  |  |  |  |  |  |  |  |  |  |  |  |  |
|  | Negative | 1 |  |  | 1 |  |  | 1 |  |  | 1 |  |  | 1 |  |  |
|  | Positive | 2.369 | 2.076-2.703 | <0.001 | 2.662 | 0.992-7.143 | 0.052 | 1.351 | 0.638-2.862 | 0.432 | 1.358 | 0.956-1.927 | 0.087 | 1.924 | 1.660-2.231 | <0.001 |
| Pathologic N stage | |  |  |  |  |  |  |  |  |  |  |  |  |  |  |  |
|  | N0 | 1 |  |  | 1 |  |  | 1 |  |  | 1 |  |  | 1 |  |  |
|  | N1 | 1.994 | 1.807-2.200 | <0.001 | 1.259 | 0.885-1.792 | 0.200 | 1.252 | 0.924-1.696 | 0.147 | 1.424 | 1.170-1.733 | <0.001 | 1.423 | 1.230-1.647 | <0.001 |
|  | N2 | 3.189 | 2.919-3.485 | <0.001 | 2.017 | 1.333-3.051 | 0.001 | 1.776 | 1.302-2.421 | <0.001 | 2.272 | 1.903-2.713 | <0.001 | 1.929 | 1.688-2.205 | <0.001 |
|  | N3 | 5.739 | 5.304-6.209 | <0.001 | 4.033 | 2.597-6.261 | <0.001 | 3.152 | 2.291-4.338 | <0.001 | 3.721 | 3.154-4.390 | <0.001 | 3.185 | 2.821-3.595 | <0.001 |
| Grade | |  |  |  |  |  |  |  |  |  |  |  |  |  |  |  |
|  | Poorly | 1 |  |  | 1 |  |  | 1 |  |  | 1 |  |  | 1 |  |  |
|  | Poorly-Moderately | 0.865 | 0.809-0.926 | <0.001 | 1.303 | 0.947-1.794 | 0.104 | 0.978 | 0.737-1.298 | 0.879 | 0.906 | 0.800-1.025 | 0.118 | 0.857 | 0.784-0.937 | 0.001 |
|  | Moderately | 0.757 | 0.703-0.815 | <0.001 | 1.521 | 1.123-2.061 | 0.007 | 0.809 | 0.597-1.095 | 0.170 | 0.740 | 0.644-0.850 | <0.001 | 0.796 | 0.720-0.879 | <0.001 |
|  | Well-Moderately | 0.488 | 0.396-0.602 | <0.001 | 1.177 | 0.682-2.030 | 0.558 | 1.292 | 0.730-2.285 | 0.379 | 0.597 | 0.374-0.953 | 0.031 | 0.478 | 0.348-0.657 | <0.001 |
|  | Well | 0.429 | 0.343-0.538 | <0.001 | 1.796 | 1.207-2.671 | 0.004 | 0.608 | 0.298-1.241 | 0.172 | 0.683 | 0.403-1.160 | 0.158 | 0.506 | 0.335-0.764 | 0.001 |
|  | Undifferentiated | 3.554 | 1.146-11.026 | 0.028 |  |  |  |  |  |  | 2.454 | 0.612-9.834 | 0.205 | 5.699 | 0.802-40.515 | 0.082 |
| Signet ring cell | |  |  |  |  |  |  |  |  |  |  |  |  |  |  |  |
|  | Yes | 1 |  |  | 1 |  |  | 1 |  |  | 1 |  |  | 1 |  |  |
|  | No | 0.91 | 0.856-0.966 | 0.002 | 1.471 | 1.148-1.885 | 0.002 | 1.185 | 0.900-1.560 | 0.228 | 0.744 | 0.664-0.834 | <0.001 | 0.769 | 0.712--0.831 | <0.001 |
| Nerve invasion | |  |  |  |  |  |  |  |  |  |  |  |  |  |  |  |
|  | Yes | 1 |  |  | 1 |  |  | 1 |  |  | 1 |  |  | 1 |  |  |
|  | No | 0.579 | 0.545-0.616 | <0.001 | 0.631 | 0.373-1.067 | 0.086 | 1.121 | 0.776-1.620 | 0.543 | 0.868 | 0.779-0.966 | 0.010 | 0.749 | 0.692-0.811 | <0.001 |
| Vascular invasion | |  |  |  |  |  |  |  |  |  |  |  |  |  |  |  |
|  | Yes | 1 |  |  | 1 |  |  | 1 |  |  | 1 |  |  | 1 |  |  |
|  | No | 0.507 | 0.480-0.536 | <0.001 | 0.754 | 0.536-1.060 | 0.104 | 0.682 | 0.532-0.876 | 0.003 | 0.653 | 0.589-0.725 | <0.001 | 0.646 | 0.601-0.695 | <0.001 |
| Linitis plastica | |  |  |  |  |  |  |  |  |  |  |  |  |  |  |  |
|  | Yes | 1 |  |  | - |  |  | 1 |  |  | 1 |  |  | 1 |  |  |
|  | No | 0.507 | 0.360-0.714 | <0.001 |  |  |  | 0.570 | 0.080-4.061 | 0.574 | 0.323 | 0.161-0.648 | 0.001 | 0.625 | 0.418-0.934 | 0.022 |
| Adjuvant therapy | |  |  |  |  |  |  |  |  |  |  |  |  |  |  |  |
|  | Yes | 1 |  |  | 1 |  |  | 1 |  |  | 1 |  |  | 1 |  |  |
|  | No | 1.872 | 1.674-2.094 | <0.001 | 0.496 | 0.344-0.717 | <0.001 | 0.741 | 0.499-1.099 | 0.136 | 1.071 | 0.859-1.335 | 0.544 | 1.198 | 1.022-1.404 | 0.026 |

Supplementary Table 2. The univariate analysis of gastrectomy patients with different ELN groups (ELN <16 and ≥16) in NCCGC cohort.

| Characteristics | | NCCGC cohort | | | T1 patients | | | T2 patients | | | T3 patients | | | T4 patients | | |
| --- | --- | --- | --- | --- | --- | --- | --- | --- | --- | --- | --- | --- | --- | --- | --- | --- |
|  |  | HR | 95%CI | P value | HR | 95%CI | P value | HR | 95%CI | P value | HR | 95%CI | P value | HR | 95%CI | P value |
| ELN | |  |  |  |  |  |  |  |  |  |  |  |  |  |  |  |
|  | <16 | 1 |  |  | 1 |  |  | 1 |  |  | 1 |  |  | 1 |  |  |
|  | ≥16 | 0.949 | 0.891-1.010 | 0.101 | 0.797 | 0.630-1.010 | 0.060 | 0.957 | 0.740-1.236 | 0.735 | 0.825 | 0.725-0.940 | 0.004 | 0.988 | 0.913-1.070 | 0.771 |
| Pathologic T stage | |  |  |  |  |  |  |  |  |  |  |  |  |  |  |  |
|  | T1 | 1 |  |  |  |  |  |  |  |  |  |  |  |  |  |  |
|  | T2 | 1.720 | 1.466-2.018 | <0.001 |  |  |  |  |  |  |  |  |  |  |  |  |
|  | T3 | 4.881 | 4.311-5.525 | <0.001 |  |  |  |  |  |  |  |  |  |  |  |  |
|  | T4 | 5.973 | 5.305-6.725 | <0.001 |  |  |  |  |  |  |  |  |  |  |  |  |
| Age at diagnosis (years) | |  |  |  |  |  |  |  |  |  |  |  |  |  |  |  |
|  | 18-34 | 1 |  |  | 1 |  |  | 1 |  |  | 1 |  |  | 1 |  |  |
|  | 35-50 | 1.057 | 0.862-1.295 | 0.595 | 2.225 | 1.440-3.438 | <0.001 | 1.164 | 0.455-2.977 | 0.751 | 0.882 | 0.562-1.383 | 0.584 | 0.925 | 0.730-1.173 | 0.522 |
|  | 51-64 | 1.142 | 0.939-1.389 | 0.185 | 7.075 | 4.647-10.773 | <0.001 | 1.564 | 0.639-3.827 | 0.328 | 0.970 | 0.628-1.498 | 0.889 | 0.861 | 0.685-1.083 | 0.201 |
|  | ≥65 | 1.770 | 1.445-2.153 | <0.001 | 0.240 | 0.033-1.773 | 0.162 | 2.960 | 1.213-7.222 | 0.017 | 1.329 | 0.860-2.054 | 0.200 | 1.174 | 0.933-1.476 | 0.171 |
| Gender | |  |  |  |  |  |  |  |  |  |  |  |  |  |  |  |
|  | Male | 1 |  |  | 1 |  |  | 1 |  |  | 1 |  |  | 1 |  |  |
|  | Female | 0.908 | 0.852-0.968 | 0.003 | 0.586 | 0.444-0.773 | <0.001 | 0.748 | 0.563-0.994 | 0.046 | 1.058 | 0.933-1.200 | 0.381 | 0.963 | 0.888-1.044 | 0.355 |
| Smoking status | |  |  |  |  |  |  |  |  |  |  |  |  |  |  |  |
|  | Smokers | 1 |  |  | 1 |  |  | 1 |  |  | 1 |  |  | 1 |  |  |
|  | Never smokers | 0.922 | 0.872-0.975 | 0.004 | 0.788 | 0.624-0.995 | 0.045 | 0.919 | 0.728-1.160 | 0.478 | 0.981 | 0.884-1.088 | 0.716 | 0.950 | 0.883-1.022 | 0.168 |
| Alcohol consumption | |  |  |  |  |  |  |  |  |  |  |  |  |  |  |  |
|  | Drinkers | 1 |  |  | 1 |  |  | 1 |  |  | 1 |  |  | 1 |  |  |
|  | Never drinkers | 0.967 | 0.912-1.026 | 0.265 | 0.994 | 0.772-1.281 | 0.963 | 0.877 | 0.687-1.120 | 0.293 | 1.019 | 0.915-1.135 | 0.726 | 0.977 | 0.906-1.055 | 0.555 |
| Location | |  |  |  |  |  |  |  |  |  |  |  |  |  |  |  |
|  | Proximal | 1 |  |  | 1 |  |  | 1 |  |  | 1 |  |  | 1 |  |  |
|  | Distal | 0.663 | 0.627-0.701 | <0.001 | 0.508 | 0.399-0.646 | <0.001 | 0.619 | 0.490-0.782 | <0.001 | 0.781 | 0.701-0.869 | <0.001 | 0.889 | 0.826-0.956 | 0.002 |
|  | Total | 1.018 | 0.864-1.198 | 0.834 | 0.751 | 0.329-1.713 | 0.496 | 0.628 | 0.307-1.285 | 0.203 | 1.045 | 0.761-1.436 | 0.783 | 1.250 | 1.018-1.535 | 0.033 |
| BMI (kg/m2) at diagnosis | |  |  |  |  |  |  |  |  |  |  |  |  |  |  |  |
|  | <18.5 | 1 |  |  | 1 |  |  | 1 |  |  | 1 |  |  | 1 |  |  |
|  | 18.5-24 | 0.849 | 0.757-0.952 | 0.005 | 1.345 | 0.747-2.423 | 0.323 | 0.457 | 0.302-0.691 | <0.001 | 0.952 | 0.750-1.207 | 0.684 | 0.872 | 0.755-1.006 | 0.061 |
|  | 24-28 | 0.731 | 0.649-0.823 | <0.001 | 1.302 | 0.717-2.363 | 0.386 | 0.391 | 0.255-0.601 | <0.001 | 0.796 | 0.623-1.015 | 0.066 | 0.793 | 0.683-0.920 | 0.002 |
|  | ＞28 | 0.672 | 0.584-0.773 | <0.001 | 0.898 | 0.453-1.781 | 0.759 | 0.320 | 0.193-0.530 | <0.001 | 0.959 | 0.729-1.260 | 0.762 | 0.738 | 0.614-0.886 | 0.001 |
| Weight loss (kg) | |  |  |  |  |  |  |  |  |  |  |  |  |  |  |  |
|  | 0-2 | 1 |  |  | 1 |  |  | 1 |  |  | 1 |  |  | 1 |  |  |
|  | 2.0-5 | 1.429 | 1.329-1.537 | <0.001 | 1.621 | 1.180-2.226 | 0.003 | 0.982 | 0.702-1.374 | 0.916 | 1.225 | 1.070-1.403 | 0.003 | 1.193 | 1.085-1.312 | <0.001 |
|  | 5-10 | 1.719 | 1.551-1.904 | <0.001 | 1.228 | 0.683-2.206 | 0.493 | 1.614 | 0.968-2.693 | 0.066 | 1.425 | 1.163-1.747 | 0.001 | 1.318 | 1.161-1.496 | <0.001 |
|  | >10 | 1.961 | 1.611-2.388 | <0.001 | 4.888 | 2.009-11.895 | <0.001 | 0.785 | 0.194-3.179 | 0.734 | 1.643 | 1.066-2.533 | 0.024 | 1.361 | 1.073-1.724 | 0.011 |
| Surgical Margin | |  |  |  |  |  |  |  |  |  |  |  |  |  |  |  |
|  | Negative | 1 |  |  | 1 |  |  | 1 |  |  | 1 |  |  | 1 |  |  |
|  | Positive | 2.322 | 2.035-2.649 | <0.001 | 2.662 | 0.992-7.143 | 0.052 | 1.351 | 0.638-2.862 | 0.432 | 1.358 | 0.956-1.927 | 0.087 | 1.924 | 1.660-2.231 | <0.001 |
| Pathologic N stage | |  |  |  |  |  |  |  |  |  |  |  |  |  |  |  |
|  | N0 | 1 |  |  | 1 |  |  | 1 |  |  | 1 |  |  | 1 |  |  |
|  | N1 | 1.941 | 1.759-2.143 | <0.001 | 1.259 | 0.885-1.792 | 0.200 | 1.252 | 0.924-1.696 | 0.147 | 1.424 | 1.170-1.733 | <0.001 | 1.423 | 1.230-1.647 | <0.001 |
|  | N2 | 3.097 | 2.833-3.385 | <0.001 | 2.017 | 1.333-3.051 | 0.001 | 1.776 | 1.302-2.421 | <0.001 | 2.272 | 1.903-2.713 | <0.001 | 1.929 | 1.688-2.205 | <0.001 |
|  | N3 | 5.556 | 5.134-6.014 | <0.001 | 4.033 | 2.597-6.261 | <0.001 | 3.152 | 2.291-4.338 | <0.001 | 3.721 | 3.154-4.390 | <0.001 | 3.185 | 2.821-3.595 | <0.001 |
| Grade | |  |  |  |  |  |  |  |  |  |  |  |  |  |  |  |
|  | Poorly | 1 |  |  | 1 |  |  | 1 |  |  | 1 |  |  | 1 |  |  |
|  | Poorly-Moderately | 0.859 | 0.803-0.920 | <0.001 | 1.303 | 0.947-1.794 | 0.104 | 0.978 | 0.737-1.298 | 0.879 | 0.906 | 0.800-1.025 | 0.118 | 0.857 | 0.784-0.937 | 0.001 |
|  | Moderately | 0.755 | 0.701-0.813 | <0.001 | 1.521 | 1.123-2.061 | 0.007 | 0.809 | 0.597-1.095 | 0.170 | 0.740 | 0.644-0.850 | <0.001 | 0.796 | 0.720-0.879 | <0.001 |
|  | Well-Moderately | 0.491 | 0.397-0.608 | <0.001 | 1.177 | 0.682-2.030 | 0.558 | 1.292 | 0.730-2.285 | 0.379 | 0.597 | 0.374-0.953 | 0.031 | 0.478 | 0.348-0.657 | <0.001 |
|  | Well | 0.435 | 0.347-0.544 | <0.001 | 1.796 | 1.207-2.671 | 0.004 | 0.608 | 0.298-1.241 | 0.172 | 0.683 | 0.403-1.160 | 0.158 | 0.506 | 0.335-0.764 | <0.001 |
|  | Undifferentiated | 3.516 | 1.133-10.907 | 0.030 |  |  |  |  |  |  | 2.454 | 0.612-9.834 | 0.205 | 5.699 | 0.802-40.515 | 0.082 |
| Signet ring cell | |  |  |  |  |  |  |  |  |  |  |  |  |  |  |  |
|  | Yes | 1 |  |  | 1 |  |  | 1 |  |  | 1 |  |  | 1 |  |  |
|  | No | 0.904 | 0.851-0.960 | 0.001 | 1.471 | 1.148-1.885 | 0.002 | 1.185 | 0.900-1.560 | 0.228 | 0.744 | 0.664-0.834 | <0.001 | 0.769 | 0.712-0.831 | <0.001 |
| Nerve invasion | |  |  |  |  |  |  |  |  |  |  |  |  |  |  |  |
|  | Yes | 1 |  |  | 1 |  |  | 1 |  |  | 1 |  |  | 1 |  |  |
|  | No | 0.589 | 0.555-0.627 | <0.001 | 0.631 | 0.373-1.067 | 0.086 | 1.121 | 0.776-1.620 | 0.543 | 0.868 | 0.779-0.966 | 0.010 | 0.749 | 0.692-0.811 | <0.001 |
| Vascular invasion | |  |  |  |  |  |  |  |  |  |  |  |  |  |  |  |
|  | Yes | 1 |  |  | 1 |  |  | 1 |  |  | 1 |  |  | 1 |  |  |
|  | No | 0.512 | 0.484-0.541 | <0.001 | 0.754 | 0.536-1.060 | 0.104 | 0.682 | 0.532-0.876 | 0.003 | 0.653 | 0.589-0.725 | <0.001 | 0.646 | 0.601-0.695 | <0.001 |
| Linitis plastica | |  |  |  |  |  |  |  |  |  |  |  |  |  |  |  |
|  | Yes | 1 |  |  | - |  |  | 1 |  |  | 1 |  |  | 1 |  |  |
|  | No | 0.517 | 0.367-0.728 | <0.001 |  |  |  | 0.570 | 0.080-4.061 | 0.574 | 0.323 | 0.161-0.648 | 0.001 | 0.625 | 0.418-0.934 | 0.022 |
| Adjuvant therapy | |  |  |  |  |  |  |  |  |  |  |  |  |  |  |  |
|  | Yes | 1 |  |  | 1 |  |  | 1 |  |  | 1 |  |  | 1 |  |  |
|  | No | 0.545 | 0.487-0.610 | <0.001 | 0.496 | 0.344-0.717 | <0.001 | 0.741 | 0.499-1.099 | 0.136 | 1.071 | 0.859-1.335 | 0.544 | 1.198 | 1.022-1.404 | 0.026 |

Supplementary Table 3. The univariate and multivariate analysis of gastrectomy patients with different ELN groups (ELN <24 and ≥24) in Northwest cohort.

| Characteristics | | Univariate analysis | | |  | Multivariate analysis | | |
| --- | --- | --- | --- | --- | --- | --- | --- | --- |
|  |  | HR | 95%CI | P value |  | HR | 95%CI | P value |
| ELN | |  |  |  |  |  |  |  |
|  | <24 | 1 |  |  |  | 1 |  |  |
|  | ≥24 | 0.958 | 0.869-1.057 | 0.393 |  | 0.754 | 0.610-0.932 | 0.009 |
| Age at diagnosis (years) | |  |  |  |  |  |  |  |
|  | 18-34 | 1 |  |  |  | 1 |  |  |
|  | 35-50 | 0.888 | 0.589-1.340 | 0.573 |  | 0.736 | 0.371-1.457 | 0.752 |
|  | 51-64 | 1.000 | 0.671-1.490 | 0.999 |  | 0.900 | 0.467-1.733 | 0.893 |
|  | ≥65 | 1.459 | 0.978-2.175 | 0.064 |  | 1.046 | 0.539-2.031 | 0.893 |
| Gender | |  |  |  |  |  |  |  |
|  | Male | 1 |  |  |  | 1 |  |  |
|  | Female | 0.858 | 0.765-0.962 | 0.009 |  | 0.746 | 0.570-0.978 | 0.034 |
| Smoking status | |  |  |  |  |  |  |  |
|  | Smokers | 1 |  |  |  | 1 |  |  |
|  | Never smokers | 1.208 | 1.058-1.379 | 0.005 |  | 0.863 | 0.651-1.145 | 0.308 |
| Alcohol consumption | |  |  |  |  |  |  |  |
|  | Drinkers | 1 |  |  |  | 1 |  |  |
|  | Never drinkers | 1.448 | 1.216-1.726 | <0.001 |  | 0.608 | 0.421-0.880 | 0.008 |
| Location | |  |  |  |  |  |  |  |
|  | Proximal | 1 |  |  |  | 1 |  |  |
|  | Distal | 0.917 | 0.807-1.042 | 0.183 |  | 0.866 | 0.677-1.106 | 0.249 |
|  | Total | 1.497 | 1.222-1.835 | <0.001 |  | 0.883 | 0.584-1.336 | 0.556 |
| BMI (kg/m2) at diagnosis | |  |  |  |  |  |  |  |
|  | <18.5 | 1 |  |  |  | 1 |  |  |
|  | 18.5-24 | 0.762 | 0.650-0.894 | 0.001 |  | 0.910 | 0.669-1.239 | 0.549 |
|  | 24-28 | 0.679 | 0.559-0.825 | <0.001 |  | 0.969 | 0.673-1.394 | 0.866 |
|  | ＞28 | 0.773 | 0.561-1.064 | 0.115 |  | 0.983 | 0.548-1.764 | 0.954 |
| Weight loss (kg) | |  |  |  |  |  |  |  |
|  | 0-2 | 1 |  |  |  | 1 |  |  |
|  | 2.0-5 | 1.159 | 1.033-1.301 | 0.012 |  | 1.035 | 0.825-1.299 | 0.766 |
|  | 5-10 | 1.747 | 1.503-2.031 | <0.001 |  | 1.263 | 0.931-1.712 | 0.133 |
|  | >10 | 2.106 | 1.583-2.802 | <0.001 |  | 1.180 | 0.615-2.264 | 0.619 |
| Surgical Margin | |  |  |  |  |  |  |  |
|  | Negative | 1 |  |  |  | 1 |  |  |
|  | Positive | 0.582 | 0.445-0.762 | <0.001 |  | 0.691 | 0.404-1.183 | 0.178 |
| Pathologic T stage | |  |  |  |  |  |  |  |
|  | T1 | 1 |  |  |  | 1 |  |  |
|  | T2 | 1.97 | 1.435-2.704 | <0.001 |  | 1.629 | 0.892-2.974 | 0.112 |
|  | T3 | 3.612 | 2.731-4.777 | <0.001 |  | 2.202 | 1.247-3.887 | 0.007 |
|  | T4 | 6.335 | 4.903-8.184 | <0.001 |  | 3.478 | 2.028-5.965 | <0.001 |
| Pathologic N stage | |  |  |  |  |  |  |  |
|  | N0 | 1 |  |  |  | 1 |  |  |
|  | N1 | 2.080 | 1.760-2.459 | <0.001 |  | 1.396 | 0.992-1.964 | 0.056 |
|  | N2 | 3.217 | 2.747-3.769 | <0.001 |  | 1.707 | 1.229-2.371 | 0.001 |
|  | N3 | 4.557 | 3.949-5.258 | <0.001 |  | 2.406 | 1.777-3.258 | <0.001 |
| Grade | |  |  |  |  |  |  |  |
|  | Poorly | 1 |  |  |  | 1 |  |  |
|  | Poorly-Moderately | 0.934 | 0.830-1.051 | 0.254 |  | 1.054 | 0.835-1.330 | 0.658 |
|  | Moderately | 0.715 | 0.626-0.817 | <0.001 |  | 1.078 | 0.795-1.461 | 0.629 |
|  | Well-Moderately | 0.535 | 0.400-0.716 | <0.001 |  | 0.871 | 0.401-1.894 | 0.728 |
|  | Well | 0.309 | 0.202-0.472 | <0.001 |  | 0.929 | 0.369-2.338 | 0.876 |
|  | Undifferentiated | 0.932 | 0.349-2.492 | 0.889 |  | 1.798 | 0.246-13.163 | 0.563 |
| Signet ring cell | |  |  |  |  |  |  |  |
|  | Yes | 1 |  |  |  | 1 |  |  |
|  | No | 0.728 | 0.643-0.824 | <0.001 |  | 0.800 | 0.612-1.045 | 0.102 |
| Nerve invasion | |  |  |  |  |  |  |  |
|  | Yes | 1 |  |  |  | 1 |  |  |
|  | No | 0.406 | 0.361-0.457 | <0.001 |  | 0.848 | 0.658-1.092 | 0.202 |
| Vascular invasion | |  |  |  |  |  |  |  |
|  | Yes | 1 |  |  |  | 1 |  |  |
|  | No | 0.410 | 0.369-0.455 | <0.001 |  | 0.584 | 0.446-0.764 | <0.001 |
| Linitis plastica | |  |  |  |  |  |  |  |
|  | Yes | 1 |  |  |  | 1 |  |  |
|  | No | 0.679 | 0.457-1.010 | 0.056 |  | 0.937 | 0.507-1.733 | 0.837 |
| Adjuvant therapy | |  |  |  |  |  |  |  |
|  | Yes | 1 |  |  |  | 1 |  |  |
|  | No | 1.095 | 0.980-1.224 | 0.110 |  | 1.587 | 1.266-1.989 | <0.001 |
